# Strategic palmar trimming before conventional shoeing shows potential for managing underrun heels in horses

**DOI:** 10.12688/f1000research.134321.1

**Published:** 2023-11-27

**Authors:** Chanoknun Poochipakorn, Kanokpan Sanigavatee, Thita Wonghanchao, Onjira Huangsaksri, Metha Chanda

**Affiliations:** 1 Veterinary Clinical Studies program, Graduate school, Kampaeng Saen Campus, Kasetsart University, Nakorn Pathom, Nakorn Pathom, Thailand; 2 Veterinary department, Thailand Equestrian Federation, Bangkok, Bangkok, Thailand; 3 Department of Large Animal and Wildlife Clinical Science, Faculty of Veterinary Medicine, Kasetsart University, Nakorn Pathom, Nakorn Pathom, Thailand

**Keywords:** heel angle, horse, strategic palmar trimming, shoeing, solar surface, underrun heels

## Abstract

**Background:** This study aimed to investigate the potential of strategic palmar trimming before conventional shoeing for managing hoof malconformation in horses with underrun heels.

**Methods:** Eleven affected horses were divided into control (5 horses with 14 defective hooves) and experimental (6 horses with 16 defective hooves) groups. The experimental group underwent strategic palmar trimming before conventional shoeing, while the control horses received regular trimming before traditional shoeing. Dorsal hoof wall angle (DHWA)/heel angle (HA) ratios, heel-bulb distance, and six parallel lines indicating solar surface dimension were determined before, and after 5, 10, 15, 20, 25, and 30 weeks of the different farriery protocols. Palmar trimming distance was only measured in experimental horses.

**Results:** A group-by-time interaction was observed for DHWA/HA ratios and solar surface dimension. Separate effects for group and time were observed in the heel–bulb distance. A modification in hoof conformation was detected at 25-30 weeks only in horses in the experimental group, including decreases in DHWA/HA ratio, heel–bulb distance, and palmar trimming distance, coinciding with an increase in palmar width of trimmed hooves.

**Conclusions:** Different trimming protocols produced a different effect on hoof conformation. Therefore, tactical remedial trimming can potentially control poor hoof conformation in horses with underrun heels.

## Introduction

Underrun heels, also known as collapsed heels or long toe–low heel conformation, are among the most common hoof distortions causing poor performance in horses ( Bigham and Tabatabaei 2007). The condition is characterised by a palmar angle that is more than 5° smaller than the hoof angle ( Dabareiner and Carter 2003, Hunt 2012, Sigafoos 1990). The hoof wall grows distally from the coronet with a uniform slope from toe to heel ( O’Grady 2008). The dorsal hoof wall angle (DHWA) and heel angle (HA) should thus theoretically be the same. However, the HA is generally smaller than the DHWA. A higher likelihood of wear at the heel than at the toe region during locomotion might contribute to angle disparity ( Barrey 1990, Moleman *et al*. 2006).

In principle, the palmar hoof region has an important role in shock absorption and management of the mechanical force applied to the hoof structure ( Eliashar 2007, Hood *et al*. 2001, O’Grady 2012). Repetitive disproportionate loading on the palmar area during movement is thought to result in underrun heels in horses ( Hunt 2012). In a severely distorted hoof, the horn tubules in the heel region are bent forward and run parallel to the ground surface ( Dabareiner and Carter 2003). As a result, this forward shift of the weight-bearing surface causes mechanical overloading of the palmar region and may generate bruising and chronic palmar pain ( Hunt 2012). Moreover, repetitive overloading, concomitant with a low heel and interphalangeal joint dorsiflexion, creates a negative angle between the distal phalanx’s solar surface and the ground ( Floyd 2010, O’Grady 2006). This negative angle may induce tissue injuries in the palmar region, thereby reducing its ability to dissipate the descending mechanical force during locomotion ( Floyd 2010, Hunt 2012). Overloading also applies abnormal forces to the navicular bone and related apparatus. This induces degenerative changes in the navicular bone, leading to navicular syndrome ( McGuigan and Wilson 2001, Wilson *et al*. 2001).

The management goal in horses with underrun heels is to establish a normal hoof capsule and increase the palmar angle, which will restore hoof function and exercise performance ( Hunt 2012, O’Grady *et al*. 2018). Appropriate hoof trimming procedures have been shown to correct a negative palmar angle accompanied by a long toe–low heel conformation ( Floyd 2010). After basic trimming, heart bar shoes, straight bar shoes, or shoeing with heel plates or wide-webbed heel shoes have been utilised to support regrowth and repair; however, the shape of these shoes make them unsuitable for long-term use in competition horses ( Hunt 2012). Elevating the heel using a shoe with a built-in wedge pad to produce a normal hoof angle has also been used to correct this defect ( Dabareiner and Carter 2003, Hunt 2012); however, this method may induce progressive overloading and lead to perpetually curved horn tubules ( Hunt 2012).

The outcomes of underrun heel correction by various corrective shoeing approaches have been inconsistent. In this study, we proposed an alternative farriery protocol for managing underrun heels: strategic palmar trimming before conventional shoeing. The objectives of the present study were as follows: first, to evaluate the potential of the alternative farriery protocol to improve hoof conformation and palmar angle, and second, to investigate the estimated time required for this proposed method to improve hoof conformation variables in horses with underrun heels.

## Methods

### Ethics approval and consent to participate

The Ethics Committee for Animal Experiments of Kasetsart University approved all procedures included in this study (ACKU64-VET-040) on June 4, 2021. Since the study was conducted as routine shoeing practice in horses under veterinary supervision. Moreover, no invasive operation was performed in this study. There was, therefore, the most negligible probability of suffering during shoeing protocol throughout the study. The owners of the animals have signed an informed consent to use their animals in this study. This article is reported inline wih Animal Research: Reporting of in vivo Experiments (ARRIVE) guidelines ( Sanigavatee 2023a).

### Animals

Eleven client-owned horses (6 geldings and 5 mares, aged 13–19 years and weighing 420–480 kg) suffering from underrun heels were studied. These horses had underrun heels as determined by a difference of at least 5° between the toe and heel angles ( Hunt 2012), which was the inclusion criterion for this study (Supplemental File 1; Table S1). They were excluded from this study if the injury or lameness occurred, causing severe lameness during the study period. Horses were recruited from equestrian riding clubs, including the Horse Lovers’ Club (13.995917138209828, 100.68551338185088), Checkmate Horse Club (13.976257409277178, 100.70805959728767), House of Horse riding club (13.812821261748075, 100.78691136835704), and Bang Phli riding school (13.597609837219945, 100.6835924548628). Affected horses were randomly divided into control (5 horses, 14 defective hooves) and experimental (6 horses, 16 defective hooves) groups. The number of horses was based on clinical cases that showed underrun heel conditions. In this study, four horses suffering from underrun heels in four hooves, six horses with underrun heels in both front hooves and a horse showing underrun heels in both real hooves ( *Extended data,* Supplementary File 1; Table S1; Sanigavatee 2023b). To categorise into control and experiment groups, four horses that showed underrun heels in four hooves were distributed evenly to the control and experiment groups. Seven horses with underrun heels on either front or rear hooves were randomly selected by drawing from a hat as a lottery method. Thus the sample size (number of underrun-heeled hooves) in the control and experiment groups was 14 and 16, respectively. There were no differences in mean DHWA/HA between groups in this study (15.09 ± 1.28 (control) vs 16.69 ± 1.43 (experiment), *p* = 0.42). Before the trial, they were conventionally shod at four- to six-week intervals and usually performed aerobic exercise during 20–50 min on 2–4 days per week. The horses were kept in their usual environment. They were housed in individual stables (4 × 4 × 6 m ^3^). Commercial pellets and Pangola hay were provided three times a day, and clean water was freely accessible. There were no therapeutic interventions before the start of the study. The horses were allowed to perform light exercise during the study if lameness was reduced to uneven gait or no foot pain.

### Experimental protocol

*Lameness examinations*

All horses underwent a full lameness examination before the beginning of the study. Lameness was evaluated by three equine practitioners and scored from 0 to 5 according to the scale outlined by the American Association of Equine Practitioners (AAEP) ( Kester 1991), in which 0 denotes no lameness and 5 indicates non-weight-bearing lameness. In brief, the horses trotted on a hard surface over a total distance of 60 m. An additional lameness evaluation was performed on all defective hooves by hoof tester compression on the solar surface area. The joint flexion test, flexor tendon palpation, and extension test were performed sequentially. Palmar digital anaesthesia using 2% lidocaine hydrochloride (L.B.S. Laboratory Ltd., Bangkok, Thailand) was performed uniaxially to localise the area of the foot pain in case lameness was present in the horse. Gait analysis was also performed before each farriery session.

*Radiographic examination*

Before the first farriery session and at 15 weeks and 30 weeks, the 30 affected hooves were radiographed in dorsopalmar, lateromedial, and 65° dorsopalmar projection to assess bone defects and internal hoof configuration. The distal phalanx/ground surface angle was also assessed radiographically (Poskom PXM-20 BT, Diagnostic Imaging Systems, Inc., SD, USA) in the affected limbs, as described previously ( Kummer *et al*. 2006).

*Strategic palmar trimming and shoeing adjustments*

**Figure 1.**
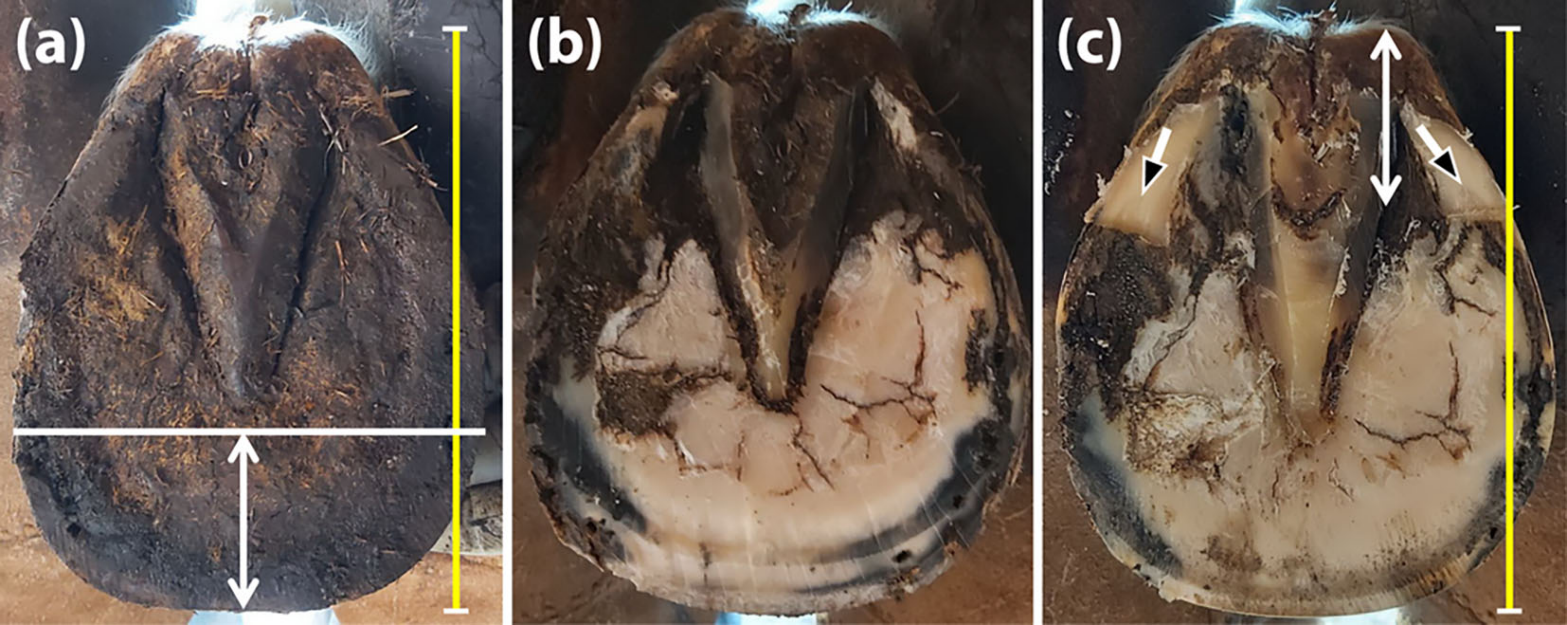
Demonstration of the solar surface of a hoof before and after trimming. The length was initially measured (yellow line) and cut at the dorsal one-third (white double-headed arrow) (a). The horn was then rasped to allow ground surface contact for approximately the dorsal two-thirds of the hoof (b). After the second measurement, the palmar third was horizontally trimmed until the sole angle was visualised (black-headed arrows). The trimming was intended to create a space between the affected palmar region and the metal shoe (c).

Each horse underwent hot-shoeing performed by experienced farriers under veterinary supervision every 5 weeks during the 30 weeks of the experiment. Of note, one horse with four defective hooves and two horses with defective front hooves were excluded from the control group due to the injury, regardless of the shoeing protocol, after 10 and 25 weeks, respectively. The strategic hoof trimming protocol in the experimental group was intended to eliminate the collapsed palmar tubules and prevent the palmar region from bearing weight. Before each farriery session, the injured hooves were measured from the toe to the heel bulb to calculate three equal areas using a Measure King™digital tool (ONTEL, Fairfield, New Jersey, USA). Each hoof was cut at the dorsal one-third and rasped to provide a ground contact surface of approximately the dorsal two-thirds of the hoof ( Figure 1a and b). The hoof was then pressed onto Play-Doh to obtain a surface model to determine the solar surface dimensions. The rasped hoof was repeatedly measured to establish the three equal regions. The palmar third of the hoof was trimmed horizontally until the angle of the sole was visualised ( Figure 1c). Finally, all horses were shod with metal shoes (Mustadfors Bruks, Dal Langed, Sweden) on the affected and healthy hooves, according to the standard method described elsewhere ( Karle *et al*. 2010, Leśniak *et al*. 2017, Weishaupt *et al*. 2013). All shoeing protocols in this study were performed closely with one experienced equine practitioner to standardise the hoof trimming method and farrier practice.

### Data acquisition and analysis

*Hoof conformation*

**Figure 2.**
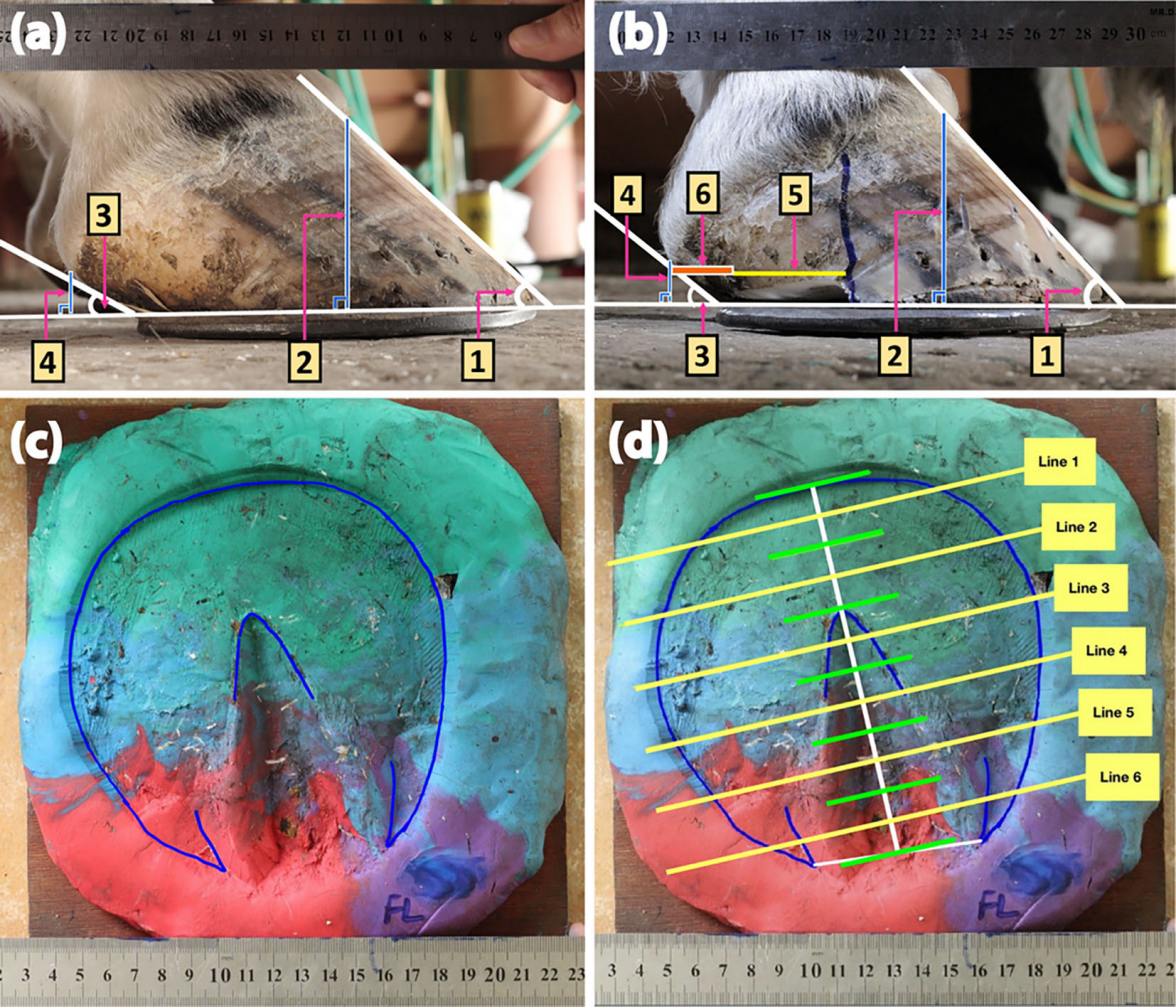
Representative images and Play-Doh models of a hoof with an underrun heel. The lateral views were captured before (a) and immediately after (b) shoeing. Measurements of the DHWA (1), hoof height (2), HA (3), heel height (4), palmar trimming distance (5), and heel-to-bulb distance (6) allowed for evaluation of the DHWA/HA ratio and the average heel–bulb and palmar trimming distances. A Play-Doh hoof model was constructed after basic trimming (c) and then divided by six parallel lines perpendicular to the central line for solar surface determination (d).

Before and after each sequential shoeing session, lateral view images of affected hooves were captured at a distance of 40 cm using a Canon EOS 500D Digital SLR Camera (Canon Inc., Tokyo, Japan) ( Figure 2a and b). The DHWA, HA, and distance of the heel bulb to the caudal ground contact of the hoof were evaluated before each shoeing session. The palmar trimming distance was estimated after each shoeing session. The data were calculated using the ImageJ program (version 1.52a) (LOCI, University of Wisconsin, Madison, WI, USA) and expressed as DHWA/HA ratio, absolute heel-bulb distance, and palmar trimming distance.

*Hoof surface dimension measurements*

The hoof surface dimensions were determined according to a method described previously ( Chanda *et al*. 2020). Briefly, Play-Doh solar surface images were captured at a constant distance of 70 cm using a Canon EOS 500D digital SLR camera (Canon Inc., Tokyo, Japan) ( Figure 2c). The circumference of the hoof ground surface was calculated using the GoodNotes 5 application (Time Base Technology Limited, Kowloon, Hong Kong). The surface was then divided by six parallel lines perpendicular to the central line of the solar surface using the AutoCAD software program (version O.49.0.0) (Autodesk Inc., San Rafael, California, USA) ( Figure 2d). Each parallel line was measured (in mm) from the medial to the lateral side using the ImageJ program (version 1.52a) (LOCI, University of Wisconsin, USA) to determine changes in the solar surface dimension.

### Statistical analyses

Statistical analyses were performed using GraphPad Prism version 9.5.1 (GraphPad Software, San Diego, CA, USA). Due to missing data in the repeated measurements, a mixed-effects model (Restricted Maximum Likelihood: REML) was used to analyse the change in the DHWA/HA ratio, absolute heel–bulb distance, and length of the six parallel lines on the solar surface in horses in the control and experimental group. Tukey’s multiple comparisons test was used to compare results within the group. Šídák’s multiple comparisons test was applied to compare parameters between the groups at given times. Palmar trimming distances were analysed by the Friedman test with a Dunn multiple comparisons test because this parameter showed a non-normal distribution following the Kolmogorov-Smirnov test. Data were expressed as the mean ± SEM. Statistical significance was set at *p* < 0.05.

## Results

### Lameness examination

The results of the lameness examination of horses in the control and experimental groups are shown in Tables 1 and 2, respectively ( Sanigavatee 2023a). Approximately half the horses exhibited mild lameness on the front legs. Furthermore, all affected horses demonstrated sensitivity to compression by the hoof tester on the sole, particularly in the palmar region. Most horses in the control group were positive for the fetlock flexion test. In contrast, only a few horses in the experimental group were positive for the fetlock flexion test. All lame horses responded to palmar digital nerve desensitisation, showing a dramatic decrease in lameness following the regional anaesthesia protocol. The radiographic images demonstrated bone defects, including synovial invagination, irregular shape of the navicular bone’s distal border, and negative palmar angle of the hoof in some horses in this study ( *Extended data,* Supplementary File 2; Sanigavatee 2023b).

**Table 1.** Lameness examination in horses in the control group before initial farriery protocols.

Hooves		Lameness examination					
		Lameness score	Hoof sensitivity test	Fetlock flexion test	Flexor tendon palpation	Carpal/hock flexion test	Extension test
Horse 1	RF	1/5 LF	++	++	–	–	–
	LF		++	++	–	–	–
Horse 2	RF	Uneven gait	+	+	–	–	–
	LF		+	+	–	–	–
Horse 3	RF	2/5 RF	++	+	–	–	–
	LF		++	+	–	–	–
	RH		+	–	–	–	–
	LH		+	–	–	–	–
Horse 4	RF	Uneven gait	+	+	–	–	–
	LF		+	+	–	–	–
	RH		+	–	–	–	–
	LH		+	–	–	–	–
Horse 5	RH	Uneven gait	+	-	–	–	–
	LH		+	-	–	–	–

LF = left forelimb; LH = left hind limb; RF = right forelimb; RH = right hind limb; **+++** = strongly positive; **++** = moderately positive; **+** = mildly positive; **–** = negative.

**Table 2.** Lameness examination in horses in the experimental group before initial farriery protocols.

Hooves		Lameness examination					
		Lameness score	Hoof sensitivity test	Fetlock flexion test	Flexor tendon palpation	Carpal/hock flexion test	Extension test
Horse 1	RF	1/5 RF	++	+	–	–	–
	LF		++	–	–	–	–
Horse 2	RF	Uneven gait	+	-	–	–	–
	LF		+	-	–	–	–
Horse 3	RF	2/5 RF	+++	–	–	–	–
	LF		++	–	–	–	–
	RH		+	–	–	–	–
	LH		+	–	–	–	–
Horse 4	RF	1/5 LF	+	+	–	–	–
	LF		+++	+	–	–	–
	RH		+	–	–	–	–
	LH		+	–	–	–	–
Horse 5	RF	No	+	-	–	–	–
	LF		+	-	–	–	–
Horse 6	RF	1/5 LF	+	-	–	–	–
	LF		++	-	–	–	–

LF = left forelimb; LH = left hind limb; RF = right forelimb; RH = right hind limb; **+++** = strongly positive; **++** = moderately positive; **+** = mildly positive; **–** = negative.

### Palmar trimming distance

**Figure 3.**
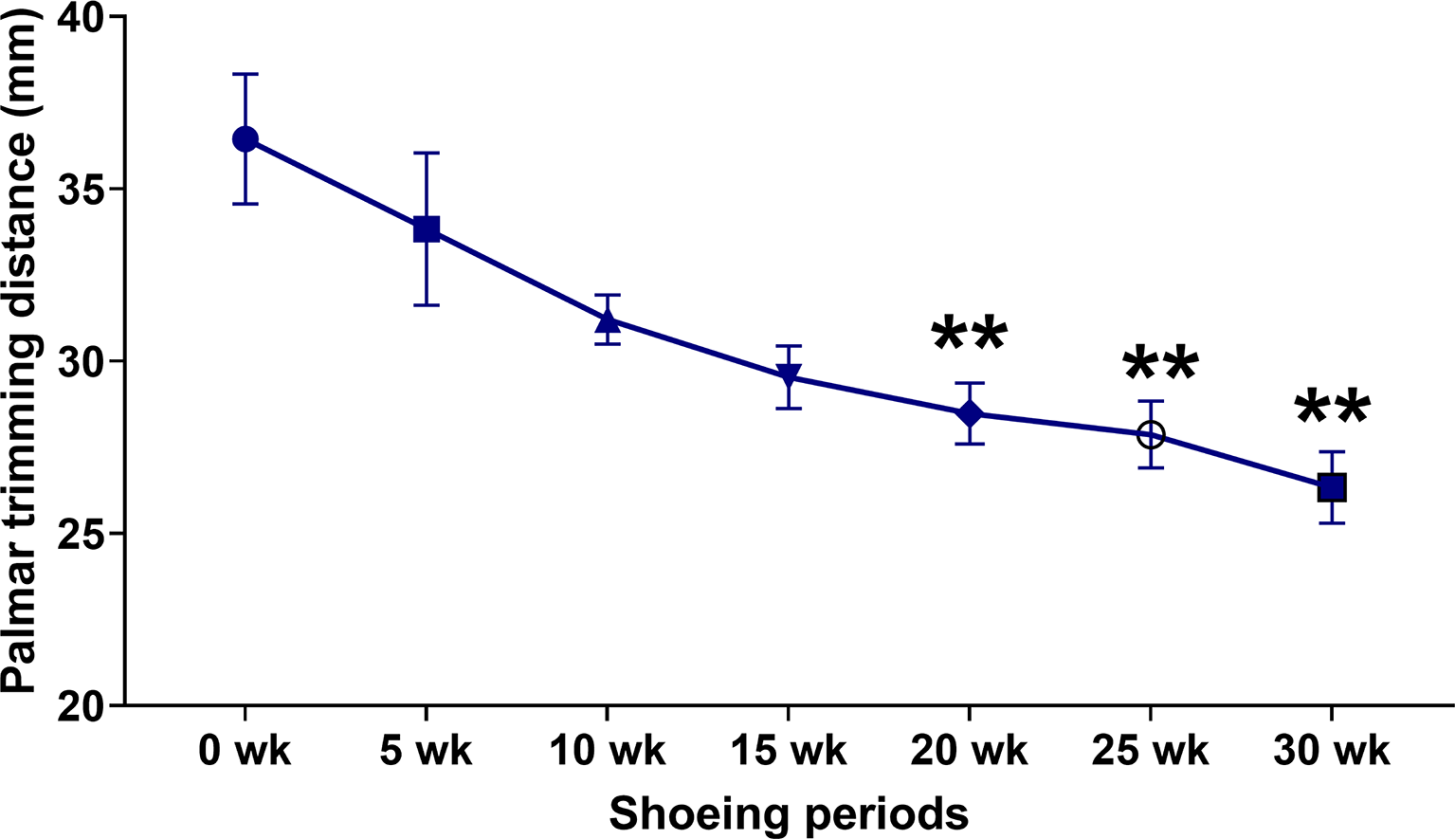
Palmar trimming distance changes in response to conventional shoeing in combination with strategic hoof trimming. The images of the front hooves of horse 2 demonstrate the palmar trimming length at the 25-week shoeing session (yellow double arrows) (a) and the 30-week shoeing session (red double arrows) (b). The line chart illustrates the quantitative change in palmar trimming distance at each shoeing session (c). ** *p* < 0.01 when compared to the values at the first shoeing session (0 wk) in horses with the strategic palmar trimming protocol.

Compared to the hooves’ palmar trimming length in the early sessions ( Figure 3a), the trimming length of the defective hooves was shorter in later shoeing sessions ( Figure 3b). A considerable shortening in palmar trimming length was detected at 25 weeks (37.51 ± 1.97 vs 27.85 ± 1.10 mm; *p* < 0.05) and 30 weeks (37.51 ± 1.97 vs 26.33 ± 1.04 mm; *p* < 0.01) ( Figure 3c).

### Heel–bulb distance

**Figure 4.**
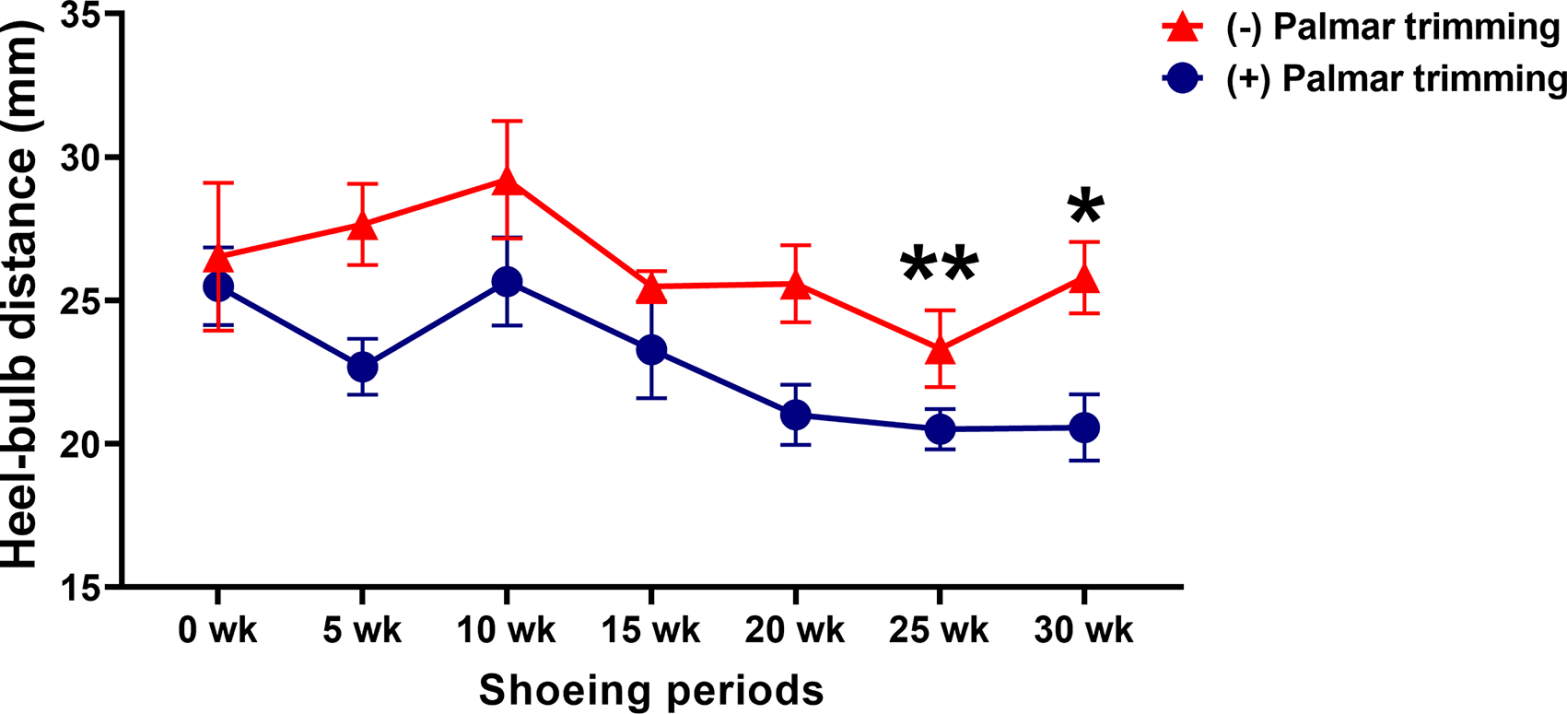
Changes in heel-bulb distance following conventional shoeing with and without strategic palmar trimming in horses with underrun heels. * *p* < 0.05 and ** *p* < 0.01 when compared to the values at the first shoeing session (0 wk) in horses with strategic palmar trimming.

Although there was no group-by-time interaction ( *p* = 0.6248), separate effects of group ( *p* = 0.0045) and time ( *p* = 0.0182) were observed for the change in the heel–bulb distance. The heel–bulb distance was unchanged in horses in the control group throughout the study period. However, in horses in the experimental group, it was significantly shorter at 25 weeks (25.50 ± 1.35 vs 20.51 ± 0.70 mm; *p* < 0.01) and 30 weeks (25.50 ± 1.35 vs 20.57 ± 1.15 mm; *p* < 0.05) ( Figure 4).

### DHWA/HA ratios

There was a group-by-time interaction ( *p* = 0.0101) for change in DHWA/HA ratios in the present study, but no separate effects of group ( *p* = 0.1208) and time ( *p* = 0.0685) were found. There was no change in DHWA/HA ratio in horses in the control group ( *Extended data,* Supplementary File 3; Figure S2a-c; Sanigavatee 2023b), but the ratio changed in the experimental group ( *Exteded data,* Supplementary File 4; Figure S3a-c; Sanigavatee 2023b).

**Figure 5.**
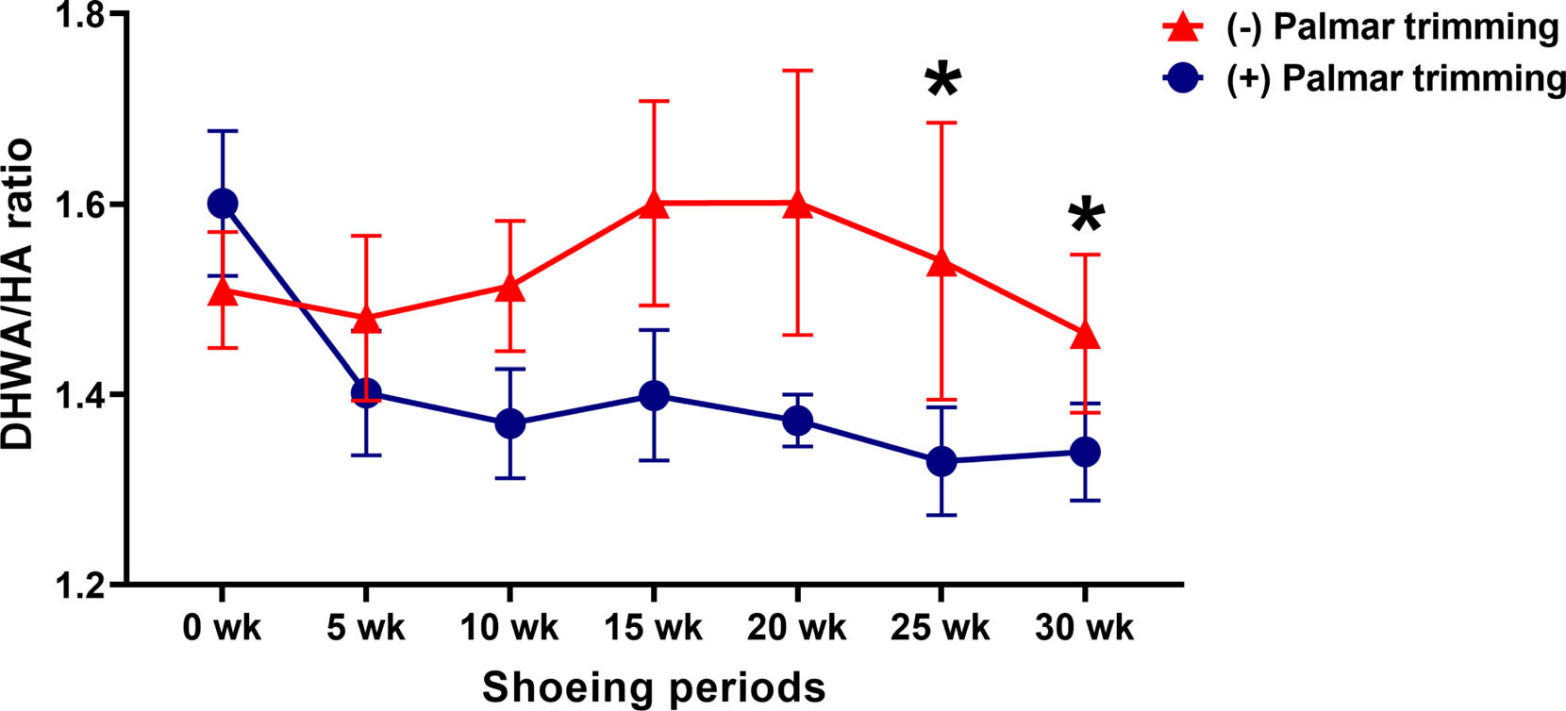
Changes in DHWA/HA angle ratio following conventional shoeing with and without strategic palmar trimming in horses with underrun heels. * *p* < 0.05 when compared to the values at the first shoeing session (0 wk) in horses with strategic palmar trimming.

A decrease in DHWA/HA ratio in experimental horses was initially detected at 25 weeks (1.60 ± 0.08 vs 1.33 ± 0.06; *p* < 0.05) and remained at 30 weeks (1.60 ± 0.08 vs 1.34 ± 0.05; *p* < 0.05) ( Figure 5). The radiographic images also showed an improved hoof angle after 30 weeks compared to the negative hoof angle at the initial farriery session in the experimental horses ( *Extended data,* Supplementary File 5; Sanigavatee 2023b).

### Solar surface dimension

Table 3 shows the modification of the solar surface, indicated by a change in the length of the six parallel lines. There was a group-by-time interaction for change in the length of all measured parallel lines (line 1; *p* = 0.0127, line 2; *p* = 0.0003, and lines 3-6; *p* < 0.0001). Time on its own interacted with a change in the length of lines 1 ( *p* = 0.0113), 3 ( *p* = 0.0202), 4 ( *p* = 0.0047), and 5 ( *p* = 0.0381), but not of lines 2 ( *p* = 0.0565) and 6 ( *p* = 0.4566). There was no separate group effect for the modification of any of the parallel lines in this study.

**Table 3.** Measurement of six parallel lines (mm) from Play-Doh impressions before each farriery session in horses with (+) and horses without (-) strategic palmar trimming.

Parallel lines	Palmar trimming	Shoeing session							Interaction *P*-value
		0 wk	5 wk	10 wk	15 wk	20 wk	25 wk	30 wk	
**Line 1**	**(-)**	80.53±2.70 ^**a**^	79.02±2.03 ^**a**^	72.56±1.46 ^**a**^	77.37±3.79 ^**a**^	78.63±3.48 ^**a**^	74.67±2.41 ^**a**^	71.63±1.60 ^**a**^	0.0127*
	**(+)**	72.77±3.31 ^**ab**^	71.79±3.14 ^**ab**^	71.89±2.88 ^**ab**^	74.19±3.20 ^**ab**^	72.29±3.00 ^**ab**^	74.86±3.62 ^**a**^	68.31±3.63 ^**b**^	
**Line 2**	**(-)**	115.59±3.49 ^**a**^	111.34±2.75 ^**a**^	105.53±2.22 ^**a**^	109.89±4.56 ^**a**^	112.91±4.13 ^**a**^	107.41±3.12 ^**a**^	102.78±1.95 ^**a**^	0.0003*
	**(+)**	106.28±4.04 ^**ab**^	105.90±3.83 ^**ab**^	109.23±3.40 ^**a**^	108.98±3.72 ^**ab**^	108.76±3.44 ^**ab**^	108.41±4.42 ^**ab**^	104.91±3.23 ^**b**^	
**Line 3** ^**‡**^	**(-)**	132.47±4.00 ^**ab**^	126.23±2.94 ^**ab**^	118.75±1.91 ^**b**^	124.82±3.97 ^**ab**^	133.23±4.61 ^**a**^	123.20±3.25 ^**ab**^	118.09±1.78 ^**b**^	<0.0001*
	**(+)**	119.46±3.90 ^**a**^	119.52±3.67 ^**a**^	122.54±3.23 ^**a**^	123.19±3.54 ^**a**^	122.51±3.15 ^**a**^	123.46±4.10 ^**a**^	119.69±2.92 ^**a**^	
**Line 4** ^**‡**^	**(-)**	132.25±4.47 ^**ab**^	126.84±2.86 ^**ab**^	121.17±1.81 ^**b**^	126.89±3.81 ^**ab**^	134.65±3.99 ^**a**^	126.66±3.14 ^**ab**^	119.60±1.96 ^**b**^	<0.0001*
	**(+)**	120.25±3.06 ^**a**^	119.63±3.03 ^**a**^	123.08±2.16 ^**a**^	123.04±2.92 ^**a**^	124.30±2.71 ^**a**^	124.27±3.47 ^**a**^	119.94±2.35 ^**a**^	
**Line 5** ^**‡**^	**(-)**	118.48±2.32 ^**a,x**^	114.01±2.42 ^**ab**^	108.54±1.96 ^**ab**^	112.78±3.62 ^**ab**^	125.82±7.96 ^**ab**^	114.23±2.67 ^**ab**^	106.38±2.29 ^**b**^	<0.0001*
	**(+)**	105.26±2.17 ^**a,y**^	106.42±2.14 ^**ab**^	109.80±1.97 ^**ab**^	109.61±2.35 ^**ab**^	109.35±2.20 ^**ab**^	111.54±2.46 ^**b**^	110.86±2.37 ^**b**^	
**Line 6**	**(-)**	87.48±2.66 ^**a,x**^	81.12±3.19 ^**a**^	78.16±3.43 ^**a**^	83.17±4.34 ^**a**^	84.16±3.71 ^**a**^	82.11±2.21 ^**a**^	78.95±2.18 ^**a**^	<0.0001*
	**(+)**	74.88±1.83 ^**a,y**^	79.08±1.87 ^**ab**^	83.55±1.68 ^**b**^	84.19±1.84 ^**b**^	83.63±2.11 ^**b**^	84.90±1.61 ^**b**^	84.74±1.24 ^**b**^	

*and ^**‡**^indicate the effects of group-by-time interaction and time on change in given parallel lines in response to palmar trimming before conventional shoeing.

The solar surface dimension was unchanged in horses in the control group throughout the study period ( *Extended data,* Supplementary File 3; Figure S2d-f; Sanigavatee 2023b). In contrast, the solar surface of the hooves in the experimental group changed over time ( *Extended data,* Supplementary File 4; Figure S3d-f; Sanigavatee 2023b). Even though a fluctuation in length was observed in lines 3 and 4 at 10, 20, and 30 weeks of the shoeing protocol (10 vs 20 weeks; *p* < 0.01 in both lines and 20 vs 30 weeks; *p* < 0.05 in both lines), the length of the six parallel lines after 30 weeks did not differ from the length before the first session in horses in the control group. Similarly, there was a marked fluctuation in the length of line 1 at 25 and 30 weeks ( *p* < 0.05) and line 2 at 10 and 30 weeks of the shoeing protocol ( *p* < 0.05) in horses in the experimental group. Nonetheless, the length of lines 1-4 at 30 weeks was not different from the value before remedial farriery.

There was a difference between groups in the length of lines 5 and 6 at the start of the trial ( *p* < 0.01 in both). A change in lines 5 and 6 was observed in the experimental group over time. The length of line 5 was increased at 25 and 30 weeks compared to the start of the trial ( *p* < 0.05 at both time points). An increase in the length of line 6 was detected earlier, at 10 weeks, until the end of the study at 30 weeks compared to before remedial farriery ( *p* < 0.01 at 10 and 15 weeks, *p* < 0.05 at 20 weeks and *p* < 0.001 at 25 and 30 weeks).

## Discussion

The present study investigated the effect of conventional shoeing combined with strategic palmar trimming on hoof conformation and recovery time in horses with underrun heels. The significant findings of the experiment include: 1) there was a farriery protocol-by-time interaction for changes in DHWA/HA ratio and solar surface dimension, indicating a distinct impact of the farriery protocol on hoof conformation; 2) hoof angle, heel angle, and solar surface dimension changed simultaneously in response to remedial farriery using strategic palmar trimming before conventional shoeing; 3) an increase in heel angle as a result of a decrease in DHWA/HA ratio was initially noted at 25 weeks; 4) a reduction in heel–bulb distance and palmar trimming length was detected at 25-30 weeks in response to remedial farriery; 5) hoof ground contact, particularly in the palmar region, improved markedly from week 25 of corrective shoeing onwards. Therefore, strategic palmar trimming followed by conventional shoeing showed potential to control underrun heels in horses. The farriery protocol should be followed for at least 25 weeks to improve hoof conformation and heel angle in horses with underrun heels.

Several pathological lesions, including palmar bruising and abscesses, sheared heels, hoof wall separation, subcapsular soft tissue damage, and osteoarthritis of the interphalangeal joints, have been reported to accompany underrun heel conformation ( Dabareiner and Carter 2003, Hunt 2012, Turner 2010, Turner 1988). Furthermore, hoof conformation defects in conjunction with the bone lesions of the navicular syndrome may be detected in horses with underrun heels ( Dabareiner and Carter 2003). This conformation defect does not always result in lameness despite repetitive abnormal loading leading to underrun heels in horses ( Hunt 2012). Moreover, the underrun heel conformation may persist without evidence of navicular syndrome ( Wright 1993). Our results support this idea, as the horses in the present study showed unilateral lameness on the front legs, even though they showed underrun heel conformation in the other limbs ( *Extended data,* Supplementary File 1; Tables S2 and S3; Sanigavatee, 2023b). Although evidence of degeneration, such as synovial invagination and irregular navicular bone shape, was not associated with lameness in the horses in this study, the mechanical force acting on the navicular bone in horses with underrun heels is still of concern ( Hunt 2012). In addition, excessive compression of the navicular bone, exerted by the tensile force of the deep digital flexor tendon, was also reported to be caused by underrun heel conformation ( Turner 1989). Hence, navicular syndrome secondary to underrun heels should still be considered.

The interval between consecutive shoeing sessions has been found to influence hoof conformation ( Leśniak *et al*. 2017, O’Grady and Poupard 2003, Van Heel *et al*. 2005). Shoeing at 8-week intervals has been reported to increase dorsal hoof wall length and decrease DHWA, which puts the distal interphalangeal joint under certain stress ( Moleman, *et al*. 2006). A 4–6-week shoeing interval could optimise the prevention of excessive loading on the palmar region, thereby lowering the risk of long-term injuries ( Leśniak *et al*. 2017). For this reason, shoeing in our recent study was carried out at five-week intervals to reap the maximum benefits of remedial farriery. Lameness in experimental horses was markedly reduced at 20–30 weeks ( *Extended data,* Supplementary File 1; Table S4; Sanigavatee 2023b), while there was almost no change in gait abnormality in the control group.

In the present study, a change in DHWA/HA ratio and heel–bulb distance was detected in horses with strategic palmar trimming before conventional shoeing, while it was unchanged in horses with regular trimming and traditional shoeing. It has been reported that appropriate trimming to restore the normal hoof angle is possibly more crucial than applying additional devices ( O’Grady 2006, O’Grady and Poupard 2001). It has been proven that hoof trimming has a significant effect on hoof conformation ( Kummer *et al*. 2006). Theoretically, hoof trimming and shoeing in a horse with foot pain should re-establish balance and correct hoof problems. Trimming and shoeing are expected to diminish the biomechanical forces exerted on the palmar hoof area ( Dabareiner and Carter 2003). Trimming and shoeing have been used to modify the external hoof capsule and the internal structures ( O’Grady 2008). Therefore, hoof trimming and shoeing are crucial in distorted hoof management ( Hunt 2012). The general concept of managing underrun heels incorporates three principles: first, toe trimming to reduce the overgrown toe, thereby facilitating breakover and restoring the proper axis and position; second, multiple rounds of palmar trimming to eliminate the curved horn tubules, creating healthy horn that can withstand palmar loading; third, proper trimming and shoeing management to reduce palmar loading on the affected hooves ( Hunt 2012). In the anatomy of the foot, the distal phalanx should reside in the dorsal two-thirds of the hoof capsule, while the soft tissues should occupy the palmar third of the hoof capsule ( O’Grady 2012). In practical terms, underrun heels may still be present even though the hoof angle has been anatomically corrected with basic hoof trimming. The palmar tubules may still be in contact with the ground, producing a fulcrum at this point when loading. This may lead to the continuous forward growth of the distorted palmar tubules, resulting in curling horn in the palmar region. The strategic palmar trimming described in the present study was designed to adhere to these three principles and enable the hooves to bear the natural descending forces. The purpose of the cut at the dorsal one-third of the hoof capsule was to shorten the toe and raise the hoof-pastern axis position. After tactical rasping, the ground contact surface extended to the dorsal two-thirds of the capsule and this was believed to be sufficient to withstand natural loading on the distal phalanx. Strategic trimming of the palmar third was designed to eliminate the curling heel and the tubules’ contact with the ground in the palmar region before shoeing. Thus, as the heels did not bear weight, the palmar horn tubules were allowed to grow distally, improving the heel angle. Moreover, this trimming protocol has considerable potential to improve the position of the axis to dissipate loading on the palmar region and promote heel growth, as demonstrated by the hoof-pastern axis improvement seen in the radiographs ( *Extended data,* Supplementary File 6; Sanigavatee 2023b). In addition, following the strategic farriery protocol, the trimming length became shorter in the palmar third while retaining an appropriate ground contact length in the dorsal two-thirds of the hoof capsule ( *Extended data,* Supplementary File 7; Sanigavatee 2023b). Lighter loading was likely experienced in the palmar region due to the strategic hoof trimming protocol. The progressive shortening of both heel–bulb length and palmar trimming distance may indicate, at least in part, that the palmar horn tubules grew more distally when this tactical shoeing protocol was applied. Therefore, the present study provided objective evidence for the importance of strategic palmar trimming in correcting underrun heels in horses.

The solar surface dimension remained constant in shod horses without strategic palmar trimming, whereas it changed over time in horses with tactical remedial trimming. The length of lines 1-4 was unchanged in horses in the present study, as the toe and quarter portions are less elastic than the heel region ( Chanda *et al*. 2020). The expansion of the palmar area, illustrated by the increased length of lines 5 and 6, was marked at 25 and 30 weeks of remedial farriery. Although the length of lines 5 and 6 was different between the two groups before the start of the trial, there was no difference between groups in the length of both lines at the end of the study: the palmar size in horses with remedial farriery increased, while it remained constant in shod horses without the strategic trimming. A decrease in heel–bulb distance and palmar trimming length, coinciding with an improvement in heel angle and other aspects of hoof conformation at 25-30 weeks of remedial farriery, suggest that this is the minimum time required to improve hoof conformation in horses with underrun heels. Nevertheless, this period may vary depending on the severity of hoof distortion, the level of experience of the farrier, the shoeing interval, and environmental factors such as riding ground surface, nutrition, and type of activity.

The small number of affected horses in each group was the main limitation of this study. Further validation of this alternative farriery protocol with a larger sample size is required. In addition, hoof radiography could not be performed at every farriery session due to lack of machine availability, so the onset of the improvement in negative palmar angle could not be documented.

## Conclusions

Strategic hoof trimming accompanied by conventional shoeing can considerably improve hoof conformation by increasing the heel angle and expanding the hooves’ palmar region in horses with underrun heels. Remedial farriery should be continued for at least 25 weeks to achieve an improvement in the affected feet. A close collaboration between a specialised veterinarian and an experienced farrier is paramount to obtain a positive outcome from strategic shoeing management in horses with underrun heels.

## Data Availability

Figshare: Data availability in horse with and without strategic palmar trimming. https://www.doi.org/10.6084/m9.figshare.23269382.v3 ( Sanigavatee 2023a). Figshare: Supplementary file: horse with and without strategic palmar trimming. https://www.doi.org/10.6084/m9.figshare.23290178.v4 ( Sanigavatee 2023b). Figshare: Completed ARRIVE checklist for ‘Strategic palmar trimming before conventional shoeing shows potential for managing underrun heels in horses’. https://www.doi.org/10.6084/m9.figshare.23269382.v3 ( Sanigavatee 2023a). Data are available under the terms of the Creative Commons Zero “No rights reserved” data waiver (CC0 1.0 Public domain dedication).
