# Supplementary material for: Strategic palmar trimming before conventional shoeing shows potential for managing underrun heels in horses
Source: F1000Res. 2024 Mar 8;12:1504. Originally published 2023 Nov 27. [Version 2] doi: 10.12688/f1000research.134321.2 (PMC11157191; doi:10.12688/f1000research.134321.2)
Supplement: Peer review history

# Reviewer report: Reviewer response for version 2

**DOI:** 10.5256/f1000research.161961.r276156

**Published:** 2024-06-06

**Authors:** Joanne Kramer

**Affiliations:** 1 Veterinary Health Center, Clydesdale Hall, Department of Veterinary Medicine and Surgery,, University of Missouri, columbia,, Missouri, USA

This study is a detailed examination of how a method of trimming protocol combined with conventional shoeing affected hooves with underrun heels over a 30 week period. A control group of 5 horses (14 affected hooves) and an experimental group of 6 horses (16 affected hooves were compared). This study draws careful conclusions supported by detailed data and results. It provides useful information regarding a common problem and I support its publication.

 Abstract-Clear. Concisely summarizes study

 Introduction-Well referenced and provides an appropriate background to the study

 Consider using the term palmar angle of the distal phalanx instead of palmar angle where appropriate

 Methods

 I am not quite clear on the  exact definition of palmar trimming distance is. From figure 2 it appears to be the horizontal distance from the start of the horizontal heel trim to the end of the heel horn  The exact definition you are using could be better clarified here so that in the results you can better comment on what made the palmar distance measurement shorter and then comment on why in the discussion

 Results

 Well presented with clear tables and figures. 

 Discussion

 Paragraph 1- How was improvement in hoof ground contact measured?  The solar surface dimension?

 This paragraph otherwise concisely summarizes the key findings

 Paragraph 2- This paragraph has some interesting points and summary statements but is the least directly related to the results and if needed for size purposes could be shortened to leave the last 3 sentences out. (okay the way it is if no space limitations)

 Paragraph 3- Well summarized

 A

 Paragraph 4- This paragraph might benefit from a sentence or two about what might be anticipated in the future for trimming after 30 weeks-expectation of ground contact with palmar heel tubules. As the heel tubule becomes straighter does this method always require trimming shorter than ground contact? Would that method of trimming be short enough for ground contact not to be achieved by the 5 week interval?  often trimming that "floats" a heel will have considerable settling even within the first week post trim.  how/why does this method differ. Also consider how this relates to number 4 in the first paragraph. What level of palmar ground contact do you want and what structures do you want loaded (eg wall tubules vs frog) 

 Paragraph 5 Well stated. see comment about palmar trimming length noted in methods

 Paragraph 6 Another limitation or at least area for future study was not evaluating frog ground contact or potentially depth and elasticity of the digital cushion between control and experimental groups or before and after strategic trimming protocol for 30 weeks.  These areas share load with the heel tubules and may affect results or conversely may be affected by the trimming protocol.  Separating fore feet from hind feet in a larger study may also be informative.

Is the work clearly and accurately presented and does it cite the current literature?

Yes

If applicable, is the statistical analysis and its interpretation appropriate?

I cannot comment. A qualified statistician is required.

Are all the source data underlying the results available to ensure full reproducibility?

Yes

Is the study design appropriate and is the work technically sound?

Yes

Are the conclusions drawn adequately supported by the results?

Yes

Are sufficient details of methods and analysis provided to allow replication by others?

Yes

Reviewer Expertise:

equine lameness

I confirm that I have read this submission and believe that I have an appropriate level of expertise to confirm that it is of an acceptable scientific standard.

# Reviewer report: Reviewer response for version 2

**DOI:** 10.5256/f1000research.161961.r254053

**Published:** 2024-03-23

**Authors:** Anderson Fernando de Souza

**Affiliations:** 1 Universidade de Sao Paulo, São Paulo, State of São Paulo, Brazil

The authors responded satisfactorily to the suggested corrections. I am now in a position to recommend acceptance of the manuscript.

Is the work clearly and accurately presented and does it cite the current literature?

Yes

If applicable, is the statistical analysis and its interpretation appropriate?

Yes

Are all the source data underlying the results available to ensure full reproducibility?

Yes

Is the study design appropriate and is the work technically sound?

Partly

Are the conclusions drawn adequately supported by the results?

Yes

Are sufficient details of methods and analysis provided to allow replication by others?

Partly

Reviewer Expertise:

Equine lameness, hoof balance, biomechanics, fracture repair, large animal surgery.

I confirm that I have read this submission and believe that I have an appropriate level of expertise to confirm that it is of an acceptable scientific standard.

# Reviewer report: Reviewer response for version 1

**DOI:** 10.5256/f1000research.147370.r229996

**Published:** 2024-01-02

**Authors:** Anderson Fernando de Souza

**Affiliations:** 1 Universidade de Sao Paulo, São Paulo, State of São Paulo, Brazil

This article describes a method for correcting the hooves of horses with underrun heels, by making specific trimming in the palmar/plantar region of the hoof associated with the use of horseshoes.

 Basically, the authors relieved the load applied to the caudal portion of the hoof in order to allow the tubules in this region to grow more vertically. There were interesting results that may support a benefit over the control group.

 Generally, the article is methodologically appropriate and relevant, despite the small number of animals evaluated.

 In this reviewer's view, the proposed method could make sense in a rigid structure, but in the horny tissue of the hoof, which is flexible, the floating effect of the heels probably lasts for a short time after hoof trimming. This may be due to deformation of the wall, loosening of the nails, and/or bending of the horseshoe, causing the part of the hoof wall that has been relieved to become load-bearing again. I would like the authors to include information and discussions about the issue of the horseshoe in relation to the hoof before re-shoeing.

 Below is a list of suggestions for the authors to consider:

 Abstract

 - Include some background or change the sub-item to "Objectives".

 - Perhaps the term "imbalance" would be more appropriate than "malconformation".

 Introduction

 - Include the study's hypothesis;

 - In the objectives, the authors refer to an "alternative farriery protocol", but in this case, the term "trimming" would be more appropriate, as this was the only difference between the groups.

 Methods

 - Use the terms "fore and hind limbs", and "fore and hind hooves" to refer to the limbs throughout the text.

 - "Real hooves"? did you mean "hind hooves"?

 - The authors included lame animals in the study and indicated that some of them improved over the course of the study. How do the authors differentiate the improvement in hoof balance from the proposed treatment and not from the simple return of the hoof load to the lame limbs?

 - The manuscript indicates that several farriers perform hoof management, but with a small number of animals, this can be an important source of variation. Ideally, only one farrier should have performed the management. Please indicate how many farriers took part in the study and whether the authors considered this a source of variation in their data analysis.

 - Three animals were removed from the control group, does this mean that only 3 animals finished the study? This reflects an important limitation for the analysis, as you probably cannot identify differences for this group. I suggest that the authors revise their statements to support their conclusions for the moments when all the animals were present in the study and only make assumptions for later moments.

 Results

 - The term "uneven gait" seems very subjective to me, what do the authors mean by this?

 - In the text there is a call for figures 3a, 3b and 3c, but there is only one figure 3. Check this.

 - Include the exact probability values throughout the text.

 - Figure 3: in the caption, there is mention of hoof images, but there is nothing of this in the figure, check this.

 - Figure 4: place the figure after the caption in the text.

 - Table 3: what do the superscript letters mean?

 Discussion

 - In the first paragraph the authors need to be clear in their statements about which groups were better in which aspects analyzed, just saying that "changes" happened is too general.

 - "Remedial farriery", "corrective shoeing"... avoid using so many terms to refer to the same subject, as it creates confusion in understanding.

Is the work clearly and accurately presented and does it cite the current literature?

Yes

If applicable, is the statistical analysis and its interpretation appropriate?

Yes

Are all the source data underlying the results available to ensure full reproducibility?

Yes

Is the study design appropriate and is the work technically sound?

Partly

Are the conclusions drawn adequately supported by the results?

Yes

Are sufficient details of methods and analysis provided to allow replication by others?

Partly

Reviewer Expertise:

Equine lameness, hoof balance, biomechanics, fracture repair, large animal surgery.

I confirm that I have read this submission and believe that I have an appropriate level of expertise to confirm that it is of an acceptable scientific standard, however I have significant reservations, as outlined above.
